# Hematopoietic-Extrinsic Cues Dictate Circadian Redistribution of Mature and Immature Hematopoietic Cells in Blood and Spleen

**DOI:** 10.3390/cells8091033

**Published:** 2019-09-05

**Authors:** Miriam Stenzinger, Darja Karpova, Christian Unterrainer, Sabine Harenkamp, Eliza Wiercinska, Keven Hoerster, Martina Pfeffer, Erik Maronde, Halvard Bonig

**Affiliations:** 1 Institute for Immunology, University Hospital Heidelberg and Institute for Clinical Transfusion Medicine and Cell Therapy, 69120 Heidelberg, Germany; 2 Institute for Transfusion Medicine and Immunohematology, Goethe University and German Red Cross Blood Service Baden-Württemberg-Hessen, Institute Frankfurt a. M.; 60528 Frankfurt a. M., Germany; 3 Division of Stem Cells and Cancer, German Cancer Research Center (DKFZ), 69120 Heidelberg, Germany; 4 Institute for Anatomy II, Division of Medicine, Heinrich Heine University, 40225 Düsseldorf, Germany; 5 Institute for Anatomy III, Goethe University, 60596 Frankfurt a. M., Germany

**Keywords:** hematopoiesis, circadian rhythm, clock genes, bmal1, CXCL12, cortisol

## Abstract

Circadian oscillations in circulating leukocyte subsets including immature hematopoietic cells have been appreciated; the origin and nature of these alterations remain elusive. Our analysis of wild-type C57BL/6 mice under constant darkness confirmed circadian fluctuations of circulating leukocytes and clonogenic cells in blood and spleen but not bone marrow. Clock gene deficient *Bmal1−/*− mice lacked this regulation. Cell cycle analyses in the different hematopoietic compartments excluded circadian changes in total cell numbers, rather favoring shifting hematopoietic cell redistribution as the underlying mechanism. Transplant chimeras demonstrate that circadian rhythms within the stroma mediate the oscillations independently of hematopoietic-intrinsic cues. We provide evidence of circadian CXCL12 regulation via clock genes in vitro and were able to confirm CXCL12 oscillation in bone marrow and blood in vivo. Our studies further implicate cortisol as the conveyor of circadian input to bone marrow stroma and mediator of the circadian leukocyte oscillation. In summary, we establish hematopoietic-extrinsic cues as causal for circadian redistribution of circulating mature/immature blood cells.

## 1. Introduction

Circadian rhythms serve to anticipate regularly occurring environmental changes, specifically light–dark cycles, in order to prepare the organism for the consequences thereof, such as changes in physical activity, food availability, and pathogen exposure [1,2]. In mammals, the circadian clock is organized in a hierarchical fashion: the suprachiasmatic nucleus (SCN) in the hypothalamus, acting as the central pacemaker, receives light input from the retina and conveys this timing information to the entire organism via subordinate oscillators in peripheral tissues [3]. In the absence of external entraining cues, so called “zeitgebers”, the intrinsic circadian system is defined as self-sustainable and persists with a period length of about 24 h under constant conditions. This feature distinguishes it from diurnal rhythms, which are extinguished under constant darkness [3]. Expression of circadian-regulated targets by definition follows a sinusoidal course over the day [1], and thus can only be established by round-the-clock analysis. Peripheral oscillators exist in many organs and are synchronized by the SCN via direct and indirect routes. Direct synchronization is mediated through humoral or neuronal signals, such as rhythmic cortisol release or autonomic nervous system innervation. Indirect entraining of peripheral oscillators is accomplished via activity–rest cycles, affecting behavior like feeding time and enabling rhythmic hormone release [1,4]. At the molecular level, the clock mechanism in central and peripheral oscillators is driven by an interlocked network of transcriptional feedback loops and consists of sets of proteins that act as positive (CLOCK, BMAL1) and negative (CRY1-2, PER1-3) transcription factors upon binding to E-box (like) enhancer elements. As one clock gene product decays, it eventually releases the promoter of the antagonistic clock gene in a continuous loop, lasting approximately 24 h, thus facilitating circadian cycles [5,6,7,8]. Loss of either branch of major clock genes results in loss of circadian rhythms [9,10,11]. In addition to the reciprocal regulation of the clock genes themselves, a wide panel of target genes is also subject to circadian regulation, the so-called circadian output genes. Transcriptome profiling in several tissues indicates that up to 40% of genes are transcribed in a circadian manner [4,12,13,14].

Daily changes in peripheral blood (PB) cell numbers in humans have been appreciated for decades [15,16,17]. More recently, these oscillations were found to be regulated in a circadian fashion and to occur in response to cues emanating from the SCN [18,19,20]. Different molecular mechanisms for transmission of the circadian information from the SCN to the periphery that mediate the oscillation of mature and immature blood cell concentrations have been discussed. Some mature blood cell lineages have been shown to display circadian gene expression, pointing towards a possible cell intrinsic function of clock genes in the hematopoietic compartment [21,22,23]. By contrast, other authors [19] implied a dominant role for bone marrow (BM) stroma cells as key regulator of white blood cell (WBC) oscillations.

In the study presented herein, we sought to further investigate the mechanisms conveying the circadian information from the SCN to the hematopoietic cells and to determine the contribution of cell intrinsic (hematopoietic) vs. extrinsic (stromal) cellular and molecular factors to circadian blood cell oscillation. Syngeneic and xenotransplant mouse models, including clock gene-deficient mice, were employed and the potential involvement of the CXCR4/CXCL12 signaling pathway as well as of cortisol were analyzed in vitro and in vivo.

## 2. Materials and Methods

### 2.1. Animals

All animal experiments reported in this manuscript were conducted in accordance with the Association for Assessment and Accreditation of Laboratory Animal Care (AAALAC) guidelines as approved by the municipal government (Darmstadt, Germany) and the institutional animal care and use committee. Mice were housed in microisolator cages at the conventional not specific pathogen-free (non-SPF) animal facility Central Experimental Unit (ZFE) at the Johann Wolfgang Goethe University Medical School or at the vivarium of the Anatomical Institute of the Goethe University Medical Center with access to water and chow ad libitum. Animals were held under cycles of 12 h light/12 h darkness (LD) or under constant darkness conditions (DD). Before experimentation, mice were adapted to LD for at least two weeks and afterwards transferred to constant darkness for at least three days for DD experiments. Lights on was defined as zeitgeber timer (ZT) 0 and lights off 12 h later as ZT12. Under DD conditions the corresponding time points were circadian time (CT) 0 and CT12. To evaluate the circadian nature, the pivotal experiments were carried out in round-the-clock analyses every three hours over a period of 24 h under LD and thereafter reproduced under DD conditions. Two-point analyses were inferred from these data to confirm circadian peak and trough values. C57BL/6 WT mice were purchased from Charles River Laboratories (Sulzfeld, Germany) and used for the experiments unless otherwise mentioned. B6.*129-Arntl<tm1Bra>/J* mice served as the clock gene deficient mouse model*; Bmal1−/−* mice were generated as described [24] and used for experiments, WT littermates (*Bmal1+/+*) served as controls. Non-obese diabetes/severe combined immunodeficiency interleukin-2 receptor common gamma chain knock-out (NSG) mice (NOD.Cg-*Prkdc^scid^Il2rg^tm1Wjl^*/SzJ), bred at the Georg–Speyer–Haus vivarium (Frankfurt, Germany), were a kind gift from Daniela Krause.

### 2.2. Human Volunteers

Two independent types of human volunteers were investigated: for analysis of spontaneous hematopoietic cell fluctuations in PB, untreated healthy volunteers had blood drawn in the a.m. and in the p.m. approximately at the reported peak and trough times. The donors were not subjected to stringent LD control. Paired pseudonymized PB samples were analyzed for WBC and CFU-C content, with permission from the Ethics Committee of Goethe University Medical Center (approval #329/10) and with written informed donor consent in accordance with the Declaration of Helsinki in its most recent revision. For isolation of human CD34+ cells, a bone marrow filter left over from a healthy donor (matched-unrelated registry donor) bone marrow harvest was used as the stem cell source for NOD/SCIDγc−/− xenotransplantation. The MUD donor underwent marrow harvests for an allogeneic recipient and left-over CD34+ cells were used with permission and authorization from the Ethics Committee (approval, see above).

### 2.3. Cells

Human CD34+ cells for humanizing NOD.SCIDγc−/− mice were immunomagnetically isolated according to manufacturer’s instructions (Miltenyi MACS system). Briefly, RBC were depleted by Ficoll density centrifugation, CD34+ cells were enriched using CD34+ CliniMACS reagent and autoMACS (both Miltenyi Biotec GmbH, Bergisch–Gladbach, Germany). Purity of CD34+ cells was 90.9% and the residual T-cell frequency was 0.18%. 

The murine NIH3T3 fibroblast cell line was engineered to express a luciferase construct under the *CXCL12* promoter alone or together with a BMAL1 expression cassette under regulation of a CMV-promoter (details see below). For analysis of the murine hematopoietic compartment, blood draws were done on awake mice from the facial vein. BM and spleen were harvested after cervical dislocation. BM cells were recovered by flushing femurs or tibias with bovine serum albumin-substituted phosphate-buffered saline (PBS–BSA 0.5%) and splenocytes were isolated by gentle blunt extrusion from the capsule and resuspension in PBS–BSA (0.5%). For most of the in vitro studies (migration, flow cytometry, colony assay), cells were washed and erythrocytes were lysed with ammonium chloride lysis buffer (Sigma–Aldrich, St Louis, MO, USA; or BD Biosciences, San Jose, CA, USA) prior to the assay performance.

### 2.4. In Vitro Assays

#### 2.4.1. Hemocytometry

In mice, complete blood count (CBC) and low-resolution differentials in the different organs were enumerated by an automatic hemocytometer (Hemavet cell counter, Drew Scientific, Waterbury, CT, USA). CBC analyses in humans were performed with the Sysmex XT-1800 hematology analyzer (Sysmex GmbH Germany, Nordersted, Germany).

#### 2.4.2. Colony Forming Unit Enumeration

Aliquots of murine BM (typically 50,000 cells), spleen (typically 500,000 cells), and lysed PB (typically the equivalent of 100 µL) were suspended in Dulbecco’s modified Eagle Medium (DMEM) + 10% fetal calf serum and incubated in duplicates in commercially available growth-factor-supplemented methyl cellulose medium for mouse colony-forming unit-culture (CFU-C) (Stem Cell Technologies, Vancouver, BC, USA or Cell Systems, Troisdorf, Germany) as described [25]. Burst-forming unit-erythroid (BFU-E), colony-forming unit granulocyte-macrophage (CFU-GM) and colony-forming unit granulocyte-erythrocyte-macrophage-megakaryocyte (CFU-GEMM or common myeloid progenitor) were enumerated after 6–8 days. Circulating human CFU-C were quantified by plating aliquots of lysed PB in commercial cytokine-replete methylcellulose media (StemMACS HSC-CFU lite with Epo, human; Miltenyi Biotec GmbH, Bergisch–Gladbach, Germany) as described [26]. CFU-C counts were assessed after 12–14 days.

#### 2.4.3. Flow Cytometry

For PB, spleen and BM cells, flow cytometry was performed with LSR Fortessa (BD, Heidelberg, Germany) and standard 4-laser-13-color setup with acquisition and analysis via FACSDiva software (BD). The following flow panels were used: 1. “Immunophenotype”: The panel contains the antibodies anti-mouse-CD3 (clone 17A2, BD), -B220 (clone RA3-6B2, eBioscience, Frankfurt am Main, Germany), -CD11b (clone M1/70, eBioscience), and -Gr1 (clone RB6-8C5, BioLegend, San Diego, CA, USA) as well as anti-mouse-CD45 (clone 30-F11, eBioscience), 7-AAD (BD) as viability marker, and anti-mouse-CXCR4 (clone 2B11, eBioscience or clone L276F12, BioLegend) to distinguish T-cells, B-cells, monocytes, and granulocytes and to quantify CXCR4 surface expression. Gating strategy was performed according to standard protocols for identification of different hematopoietic subpopulations [27]. Frequencies were multiplied by concentration (with Hemavet WBC count corresponding to the CD45+ fraction) to calculate total cell numbers. 2. “Hematopoietic stem and progenitor cells” (HSPC): HSPC were recognized using anti-mouse–Lin (product # 51-9003632, BD), -Ly-6A/E (sca-1, clone E13-161.7, BD), -CD117 (clone ACK2, eBioscience), -CD45 (see above), and -CXCR4 (see above) antibodies and established multicolor staining panels [28]. Frequencies among CD45+ cells were multiplied by WBC concentration (Hemavet) to calculate cell numbers per tissue. 3. “Cell cycle”: Two different facets of cell cycle activity were assessed. Cell cycle status was analyzed by intracellular Ki67/7-AAD staining, whereas recent cell cycle history was estimated by short-term BrdU-pulsing and anti-BrdU staining. For the former, after surface staining for relevant surface markers (see above) cells were fixed/permeabilized according to the manufacturer’s handbook (Cytofix/Cytoperm Plus Kit, eBioscience) and stained with Ki67/7-AAD (anti-mouse/rat Ki-67, clone SolA15, eBioscience; 7-AAD, # 51-68981E, BD) to distinguish G0, G1, and G2/S/M cell cycle phases. For the latter, cells were processed with the BrdU staining kit (FITC BrdU Flow Kit, # 559619, BD) according to the manufacturer’s instructions as described [25]. Cells from not BrdU pulsed animals were processed equally and served as gating controls. Cells which had cycled within the last three hours were recognized as BrdU positive. 

In humanized mouse blood, relative frequencies of murine and human WBC were distinguished by flow cytometry with mutually exclusive anti-muCD45 (see above) and anti-huCD45 antibodies (clone 2D1, BD), total WBC concentration was assessed by Hemavet, and total murine vs. human WBC were calculated there from.

#### 2.4.4. Transwell Migration

Migration of BM cells through 5 µm transwells (Corning–Costar, Tewksbury, MA, USA) towards CXCL12 (50 ng/mL, Peprotech, Rocky Hill, NJ, USA or Cell Systems, Kirkland, WA, USA) or control medium (spontaneous migration), was performed as described [25,27] and assessed after four hours to compare chemotaxis activity of murine BM cells towards CXCL12 at ZT2 versus ZT14.

#### 2.4.5. CXCL12 Protein Quantification

Commercially available enzyme-linked immunosorbent assay (ELISA) (Mouse CXCL12/SDF-1 alpha Quantikine ELISA Kit, R&D Systems, Minneapolis, MN, USA) was used to quantify CXCL12 protein concentrations in murine BM and PB plasma, according to the manufacturer’s handbook.

#### 2.4.6. Cortisol Quantification

PB plasma cortisol levels were measured using a commercial electro-chemoluminescence immunoassay (Cortisol II assay on cobas 6000, e Modul 602, Roche Diagnostics, Basel, Switzerland) according to the manufacturer’s instructions.

#### 2.4.7. Luciferase Reporter-Gene Assays

To test the regulation of CXCL12 expression by clock genes, a 1515 base pair fragment of the *CXCL12* promoter (chr10:44384980-44386494) was amplified from human PB WBC genomic DNA and cloned into the luciferase reporter plasmid pGL4.19[*luc2CP*/Neo] (Promega, Fitchburg, WI, USA). The expression construct was transfected into NIH3T3 cells. As a control, BMAL1 driven by a constitutive CMV-promoter was co-overexpressed with the luciferase construct in order to override cyclical BMAL1 expression in the target cells. Transfection was performed as described [29,30,31]. Endogenous cell clocks were synchronized with 1 µM dexamethasone added to the medium [32,33] and reporter gene activity kinetics were followed over a period of 120 h under static culture conditions using a microtiter plate luminometer (Lumistar; BMG, Germany) as previously described [29,30,31]. 

### 2.5. Animal Experiments

#### 2.5.1. Enforced Stem Cell Egress

A single dose of a mild pharmacological “mobilizing agent”, the CXCR4 antagonist AMD3100 (5 mg/kg, Sigma–Aldrich, St. Louis, MO, USA) was administered i.p. at ZT1 or ZT13, one hour before the expected peak or trough of CFU-C content in PB of WT mice as previously described [25]. To assess the hematopoietic cell egress, blood was drawn one hour after injection at ZT2 vs. ZT14 for WBC and CFU-C enumeration (see above).

#### 2.5.2. Cortisol Administration

Prednisolone (Sigma–Aldrich) was dissolved in DMSO and diluted in NaCl 0.9% as described [34] to obtain the cortisol injection solution (containing 1% DMSO). A single dose of 125 µg cortisol per mouse was injected s.c. in *Bmal1^−/−^* mice at ZT11, the physiological peak time point for plasma cortisol. The same dose was injected anti-cyclically at ZT0 in WT mice. In either case the administered cortisol dose aimed to achieve plasma levels approximating the endogenous cortisol peak. Control mice received prednisolone-free 1%-DMSO/NaCl solution. WBC and CFU-C count (see above) were assessed every three hours over a period of 24 h.

#### 2.5.3. Bromodeoxyuridine (BrdU) Labeling

BrdU (Sigma–Aldrich) at a dose of 0.167 mg/g or NaCl 0.9% (control) was injected i.p. into WT mice as described [25] three hours before the expected peak or trough of PB CFU-C counts at ZT23 or ZT11. Mice were sacrificed at ZT2 or ZT14 and PB, and spleen and BM cells were harvested and processed for flow cytometry analysis of BrdU incorporation (see above).

### 2.6. Splenectomy

Splenectomy was performed aseptically on anesthetized WT mice as described [35]. Mice were allowed a minimum of four weeks to recover before undergoing experiments. 

### 2.7. Transplantation Experiments

To study the effect of clock gene deficiency in the hematopoietic system versus in the stroma, 10–12 week old WT or *Bmal1−/−* hosts were irradiated with a single 950 cGy radiation dose from a solid cesium source at a dose rate of 70 cGy/min. Transplants of 2 × 10^6^ WT or *Bmal1−/−* BM cells were injected intravenously in a volume of 200 µL PBS within two hours of irradiation. After transplantation, mice received antibiotic-supplemented drinking water for two weeks for sepsis prophylaxis. Engraftment was assessed in PB 12 weeks after transplantation via qPCR analysis. Sixteen weeks post transplantation, cohorts of mice were examined for WBC and CFU-C content in PB.

Humanized mice were generated using NSG recipients. After irradiation with 100 cGy, mice received transplants of 500,000 human CD34+ cells with 1000 contaminating CD3+ cells. A single cohort, all transplanted from a single donor, was tested. Mice were kept on antibiotics for two weeks after transplantation in conventional filter-top cages. Ten weeks after transplantation, PB was analyzed for the relative contribution as well as absolute numbers of murine and human cells at peak and trough. Mice were engrafted to a human WBC content of ca. 2.5% (1000/µL murine and 25/µL human WBC).

### 2.8. Statistics

Descriptive statistics and Student’s t-tests were calculated in Excel 2010 (Microsoft, Redmond, WA, USA). Data approximation with a sinusoid function was performed assuming circadian rhythm with a 24 h period and applying cosinor analysis as described [36]. R was used as statistical software with the help of the packages cosinor and cosinor2 [37,38,39]. The shown sinusoidal functions are the best fitting sinusoids based on the least squares method. Rhythm analysis was performed by testing the null hypothesis of zero amplitude using the F-test. Both analysis tools (fitting based on least squares method, zero amplitude test) were performed as previously reported [40]. Estimates for the *midline estimating statistic of rhythm* (MESOR), amplitude, and acrophase based on the cosinor analysis are shown in Table 1. Because of the large number of tests (*n* = 26), *p*-values were calculated using the Benjamini–Hochberg correction. The applied significance level was 0.05. Up to 4 digits of *p*-values were shown to account for the Benjamini–Hochberg correction. Significant *p*-values are marked with * if *p* < 0.05, ** if *p* < 0.01, or *** if *p* < 0.001. The *n* for each experiment is indicated in the figure legends. Unless otherwise specified, all values are presented as mean ± SEM. 

## 3. Results

### 3.1. Mature and Immature Hematopoietic Cell Concentrations in Peripheral Blood and Spleen, but not Bone Marrow, are Subject to Circadian Rhythmicity

**Figure 1:**
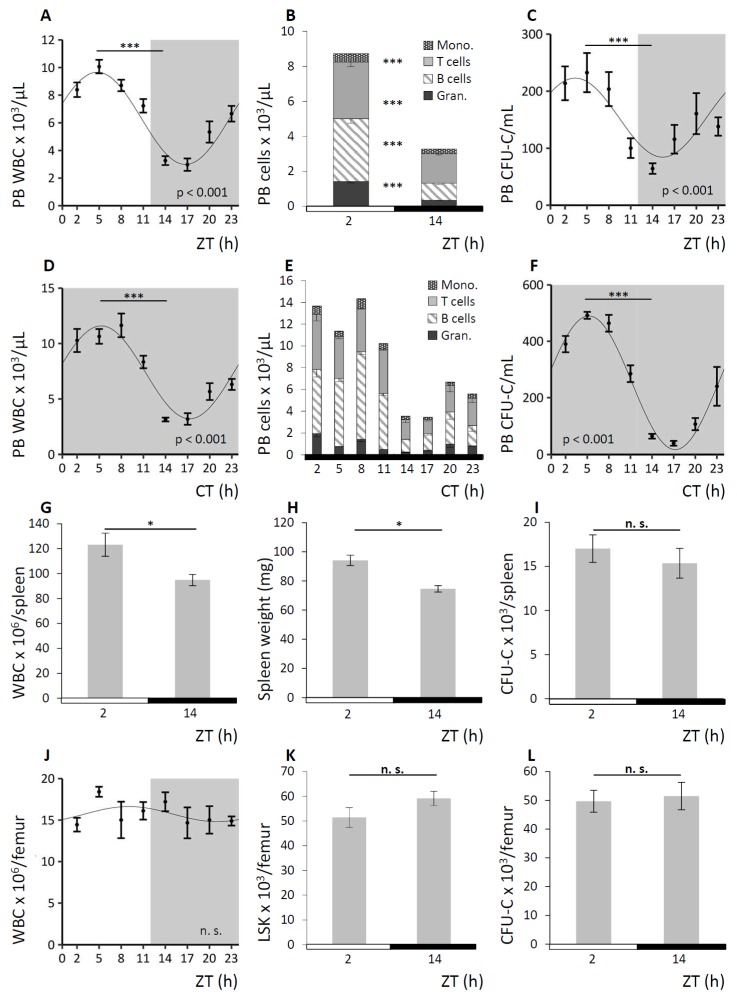
Hematopoietic cell concentrations in peripheral blood (PB) and spleen, but not bone marrow (BM), oscillate in a circadian fashion. Wild-type (WT) mice were kept under conditions of 12 h light/12 h darkness (LD) indicated by half white/half grey backgrounds or half white/half black bars below the graphs and under constant darkness conditions (DD) displayed by grey backgrounds or solid black bars below the graphs. Cosinor analysis of circadian rhythms was performed for all 24 h experiments (see Table 1 for rhythm characteristics). Mice were and analyzed for mature and immature cell contents in PB (*n* = 8–16 mice per time point), spleen (*n* = 14 mice per time point), and BM (*n* = 10 mice per time point). **A–C.** PB was assessed under LD conditions for white blood cell (WBC) count (**A**), leukocyte subpopulations (**B**), and CFU-C concentrations (**C**). **D–F.** PB was analyzed under DD conditions for WBC count (**D**), leukocyte subpopulations (**E**), and CFU-C concentrations (**F**). **G–I.** Splenic WBC counts (**G**), spleen weight (**H**), and CFU-C content (**I**) were determined at zeitgeber time (ZT) 2 and ZT14. **J–L.** BM WBC counts over time (**J**) as well as LSK (**K**) and CFU-C content per femur (**L**) at ZT2 and ZT4 were assessed. Asterisks: * *p* < 0.05, *** *p* < 0.001.

C57BL/6 wild-type (WT) mice were held under 12 h light/12 h darkness cycles (LD) or, to confirm the circadian nature of some key observations, under conditions of constant darkness (DD) as depicted in each figure. LD is indicated by half white/half grey backgrounds or half white/half black bars below the graphs, DD is displayed by grey backgrounds or solid black bars below the graphs. PB, spleen, and BM were analyzed for mature and immature blood cell content every three hours for a period of 24 h. A summary of the observations under LD conditions is shown in Figure 1, results obtained under DD conditions are exemplarily shown for PB analyses. In PB, WBC counts varied four-fold with numbers following a significant sinusoidal function under LD (Figure 1A). Circadian nature of the WBC oscillation was confirmed under DD conditions (Figure 1D). The WBC acrophase was detected in the morning (corresponding to ZT2-5) at the beginning of the resting phase, and trough cell numbers (nadir) were measured about 12 h later between ZT14-17, with the beginning of the active phase of mice. The observed oscillations affected all mature lineages to a similar degree (Figure 1B for LD and Figure 1E for DD) and circadian rhythmicity was confirmed for all subpopulations under DD conditions (Figure S1). Following a similar pattern, although even more pronounced, a significant sinusoidal function with 10-fold differences in circulating clonogenic cell (CFU-C) counts was observed over the course of the day under LD (Figure 1C) and circadian regulation of the CFU-Cs was assessed under DD conditions (Figure 1F). In spleen, total WBC and contributing lineages as well as immature cells approximately reflected the changes in PB, with significantly increased matutinal WBC content (Figure 1G) and spleen weight (Figure 1H) but not CFU-C numbers per spleen (Figure 1I). By contrast, mature and immature BM cellularity did not change noticeably over the course of the day, i.e., WBC (Figure 1J) and corresponding subpopulations, Lin^-^Sca1^+^Kit^−^ (LSK) cell frequency (Figure 1K), and CFU-C number (Figure 1L) in BM were not subject to detectable circadian variation. However, given the abundance of cells in the BM compared to the peripheral blood system, small changes in BM content could account for the observed peripheral blood cell changes.

**Figure 2:**
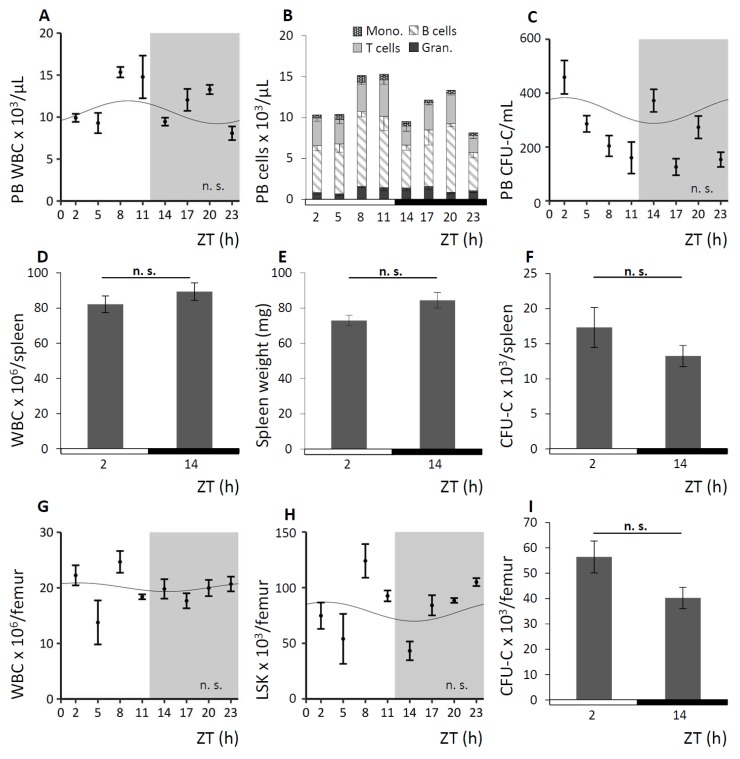
*Bmal1−/−* mice lack circadian blood cell oscillation**.** *Bmal1−/−* mice were kept under LD or DD conditions, Figure 2 exemplarily shows experiments under LD. Cosinor analysis of circadian rhythms was performed for all 24 h experiments (see Table 1 for rhythm characteristics). Mice were analyzed for mature and immature blood cell contents in PB (*n* = 5–15 mice per time point), spleen (*n* = 6 mice per time point), and BM (*n* = 5–9 mice per time point). **A–C.** PB WBC (**A**), leukocyte subpopulations (**B**) and CFU-C concentrations (**C**) were assessed over time. **D–F.** Splenic WBC (**D**) and CFU-C content (**F**) as well as spleen weight (**E**) were determined at ZT2 and ZT14. **G–I.** BM WBC (**G**) and LSK content (**H**) over time as well as CFU-C concentrations (**I**) at ZT2 and ZT14 were assessed.

To further verify the circadian nature of the rhythm guiding the oscillation of circulating cells, we took advantage of the *Bmal1*-deficient (*Bmal1−/−*) mouse model. Deficiency for the clock gene *Bmal1* had been reported to result in a complete loss of circadian rhythmicity and to abolish diurnal hematopoietic cell fluctuations [9]. Significantly expanding on previous observations [19], round-the-clock analyses of the hematopoietic compartments under LD and DD conditions were performed and results of the observation under LD conditions are shown in Figure 2. As expected, analyses of *Bmal1−/−* mice revealed a complete lack of rhythmicity in the number of circulating mature and immature hematopoietic cells in PB (Figure 2A–C) of all subpopulations. We further established the absence of circadian rhythms in spleen (Figure 2D–F) and BM (Figure 2G–I).

Apart from the apparent loss of circadian rhythmicity, hematopoiesis in *Bmal1−/−* mice was functional and unremarkable except for a slightly higher WBC cell count in PB compared to littermate WT in line with previously reported data [41]. The slightly higher amounts of mature and immature hematopoietic cells in PB remain within what is considered a normal range for C57Bl/6 mice and hematopoietic lineages were found in normal distribution in this mouse strain.

### 3.2. Hematopoietic Cell Oscillations upon Enforced Stem Cell Mobilization

**Figure 3:**
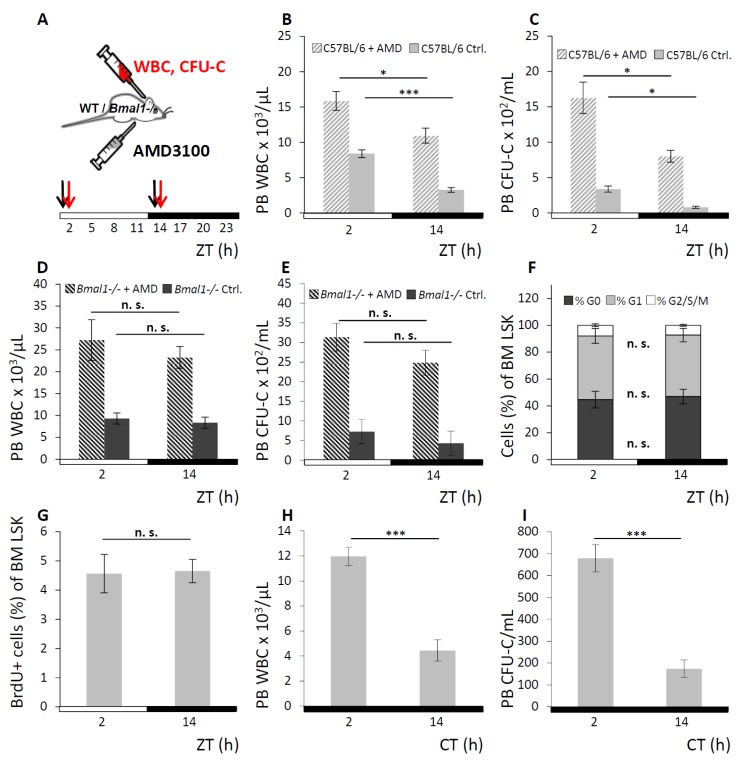
Augmented circadian hematopoietic cell oscillation under AMD3100. **A.** Scheme of the AMD3100 mobilization experiment: WT and *Bmal1−/−* mice were held under LD conditions and received i.p. injections of AMD3100 at ZT1 and ZT13 (black arrows). PB was collected at ZT2 and ZT14 (red arrows) and analyzed for leukocyte content. **B, C.** WT mice received AMD3100 (hatched bars) or saline injection (solid grey bars) and were analyzed for circulating WBC (**B**) and CFU-C counts (**C**) with *n* = 9 mice per each group and time point. **D, E.** *Bmal1−/−* mice received injections of AMD3100 (hatched bars) or saline (dark grey bars), followed by WBC (**D**) and CFU-C enumeration (**E**), *n* = 6 mice per each group and time point. **F–I.** Circadian oscillation of hematopoietic cells is due to differential cell distribution. **F, G.** Cell cycle activity of BM LSK cells was assessed by Ki67/7-AAD co-staining (**F**, G0 phase depicted in dark grey, G1 phase in light grey and G2/S/M phase in white) or BrdU incorporation (**G**) at ZT2 and ZT14 after 3-h BrdU pulsing (*n* = 6 mice per time point). **H, I.** Splenectomized WT mice were kept under DD conditions and analyzed for PB WBC (**H**) and CFU-C content (**I**) at CT2 or CT14 (*n* = 5 mice per time point). Asterisks: * *p* < 0.05, *** *p* < 0.001.

We next assessed whether the enforced egress of WBC and CFU-C in response to pharmacological “mobilizing agents” would be affected by the endogenous rhythmicity of hematopoietic cell fluctuation, as was previously suggested [42]. A bolus of AMD3100 was injected in WT or *Bmal1−/−* mice one hour before the expected physiological leukocyte peak or trough at ZT1 or ZT13 respectively. Control mice received saline injections at the same time points. AMD3100 mobilization response was assessed at ZT2 vs. ZT14 (for experimental set-up see Figure 3A). In WT mice, significantly higher numbers of circulating WBC (Figure 3B) and CFU-C (Figure 3C) were found when mobilization occurred in the morning than in the evening, which affected all mature and immature subpopulations (Figures S2 and S3). By contrast, in *Bmal1−/−* mice the AMD3100 induced leukocyte egress was not appreciably different at ZT2 vs. ZT14 for WBC (Figure 3D) and CFU-C counts (Figure 3E), although mobilization was overall more efficient in *Bmal1−/−* than in WT mice.

### 3.3. Circadian Oscillation of Hematopoietic Cells is Due to Recirculation between Blood/Spleen and Other Physical Sites

To examine whether bursts of newly emerged cells as opposed to their redistribution represent the source of the observed blood cell fluctuations, cell cycle status and history were determined. Analyses of mature and immature leukocytes from PB, spleen, and BM of WT mice were performed at the time points preceding physiological peak and trough of PB WBC counts. Cell cycle phase distribution was analyzed by Ki67/7-AAD co-staining, and recent cell cycle activity was estimated by short-term BrdU pulsing and staining. No circadian variation of the cell cycle distribution within mature (Lin+) or immature (LSK) hematopoietic cells was found in either of the three hematopoietic tissues, exemplarily shown for BM LSK cells (Ki67/7-AAD, Figure 3F; 3-h BrdU-incorporation, Figure 3G; for FACS gating strategy see Figure S4). The same outcomes were observed in Lin+ BM cells (for Ki67/7-AAD see Figure S5; for 3-h BrdU-incorporation see Figure S6) as well as in PB and spleen (data not shown). This argues in favor of redistribution between the tissues rather than altered proliferation as the mechanism responsible for changes in peripheral leukocyte numbers.

Possible contribution of spleen derived cells to oscillations in PB was assessed in splenectomized WT mice. Matutinal versus vespertine PB WBC (Figure 3H) and CFU-C contents (Figure 3I) were measured and rhythmicity was found to be maintained. These data contradict redistribution from the spleen as a potential origin of the circadian blood cell oscillation and instead point towards the BM as the organ from where mature and immature hematopoietic cells are rhythmically released.

### 3.4. Hematopoietic-Extrinsic Cues Dictate the Rhythmical Circadian Fluctuation of Hematopoietic Cells

**Figure 4:**
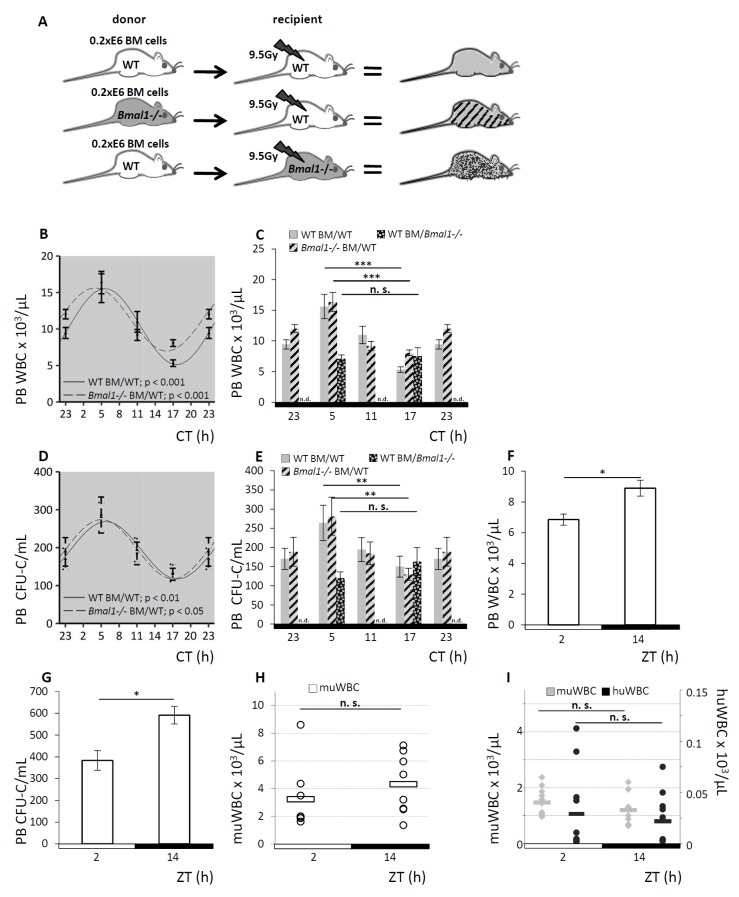
Hematopoietic-extrinsic cues dictate the rhythmical circadian fluctuation of hematopoietic cells**. A.** Scheme of the experimental set-up: radiation chimeras were generated by transplanting WT BM cells to WT hosts (grey, *n* = 12 per time point), *Bmal1−/−* BM to WT hosts (hatched, *n* = 12 per time point) or WT BM to *Bmal1−/−* hosts (spotted black, *n* = 12 per time point). After reconstitution, mice were held under DD conditions and PB WBC and CFU-C content was determined over time. **B.** Cosinor analysis was performed for PB WBC of WT host reconstituted with WT BM (solid line) or *Bmal1−/−* BM (dashed line), see Table 1 for circadian rhythm characteristics. **C.** PB WBC counts shown as bar graphs, grey bars indicate WT mice reconstituted with WT hematopoiesis, hatched bars represent WT mice reconstituted with *Bmal1−/−* BM, and spotted black bars depict *Bmal1−/−* recipients reconstituted with WT BM. **D.** Cosinor analysis was performed for PB CFU-C of WT host reconstituted with WT BM (solid line) or *Bmal1−/−* BM (dashed line), rhythm parameters are listed in Table 1. **E.** PB CFU-C counts shown as bar graphs, grey bars indicate WT mice reconstituted with WT hematopoiesis, hatched bars represent WT mice reconstituted with *Bmal1−/−* BM and spotted black bars depict *Bmal1−/−* recipients reconstituted with WT BM. **F, G.** PB from human volunteers (*n* = 6) was analyzed at ZT2 or ZT14 for PB WBC (**F**) and CFU-C content (**G**). **H.** NSG mice were analyzed for PB WBC counts at ZT2 and ZT14 (*n* = 8 mice per time point). **I.** In humanized NSG mice, human (black dots) and murine WBC counts (grey dots) were enumerated at ZT2 and ZT14, 10–12 weeks after transplantation (*n* = 12 mice per time point, each dot represents a single measurement in one mouse). Asterisks: * *p* < 0.05, ** *p* < 0.01, *** *p* < 0.001.

To further address the source of the circadian rhythm guiding the redistribution of circulating blood cells—the hematopoietic cell proper versus its environment—we again took advantage of the clock gene-deficient *Bmal1−/−* mouse model. We generated radiation chimeras with WT hematopoiesis in an otherwise *Bmal1*-deficient environment vs. *Bmal1−/−* hematopoiesis in WT recipients. WT hematopoiesis in a WT environment served as control. A scheme of the experimental set-up is shown in Figure 4A. Full donor chimerism was confirmed 12 weeks after transplantation by qPCR genotyping of PB leukocytes.

In WT recipients, irrespective of the repopulating bone marrow’s genotype, *Bmal1−/−* or WT, analyses of PB WBC and CFU-C after hematopoietic reconstitution reproduced significant sinusoidal rhythmicity which persisted under DD conditions (Figure 4B,C for PB WBC counts, Figure 4D,4E for PB CFU-C content). Both groups displayed an identical pattern of matutinal acrophases of WBC and CFU-C numbers in the circulation, followed by nadirs 12 h later. The range of the leukocyte counts and sinusoidal curve progression was comparable to untreated WT mice (see Figure 1).

By contrast, the WT hematopoietic cells in *Bmal1−/−* hosts lacked sinusoidal WBC (Figure 4C) and CFU-C oscillation (Figure 4E) and were completely arrhythmic, just as observed in pure clock gene deficient mice (see Figure 2). 

These results demonstrate that the endogenous clock within the hematopoietic cells is entirely dispensable for the circadian blood cell oscillation as the clock gene deficient *Bmal1−/−* hematopoiesis adapts to the physiological circadian rhythm in a WT environment. On the contrary, circadian regulated hematopoietic-extrinsic factors are responsible for the rhythmic redistribution of mature and immature blood cells.

Exploratory analyses in human volunteers showed diurnal variation in WBC (Figure 4F) and CFU-C (Figure 4G) in the circulation, similar, albeit inverted relative to mice, with the nadir in the morning and peak values in the evening.

To further corroborate the effect of hematopoietic-extrinsic cues on the circadian blood cell oscillation, we investigated the xenotolerant complement-deficient NOD/SCID common-gamma-chain-deleted (NSG) mouse model. Given that the distal part of the complement cascade was previously identified as a critical component of blood cell circadian rhythms [43], the expectation was to observe markedly attenuated hematopoietic rhythmicity and indeed this was the case: PB WBC counts lacked circadian variation (Figure 4H), as well as human cells engrafted in these mice lost appreciable circadian rhythmicity (Figure 4I). These observations confirm hematopoietic-extrinsic cues as the underlying mechanism for the observed circadian hematopoietic cell redistribution.

### 3.5. CXCL12 Underlies Circadian Regulation In Vitro and In Vivo

**Figure 5:**
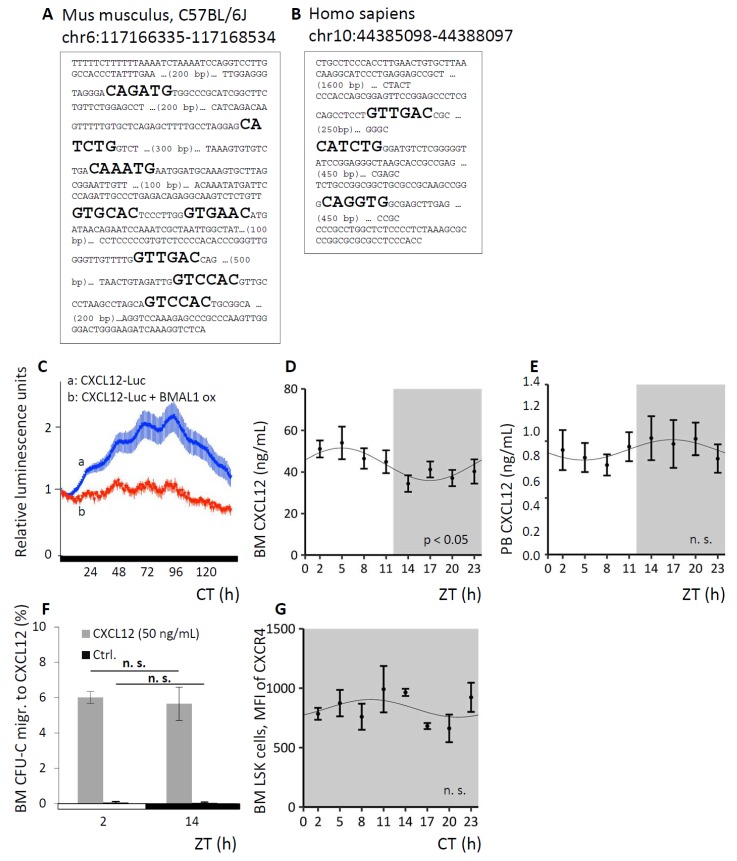
Circadian regulation of CXCL12. **A, B.** In silico analyses identified 8 E-boxes in the murine (**A**) and 3 E-boxes in the human *CXCl12* promoter (**B**). **C.** Luciferase expression in NIH3T3 cells under control of the *CXCL12* promoter reveals circadian regulation of the promoter activity after cell synchronization with dexamethasone (**a**). Co-transfection of the *CXCL12*-luc construct with a BMAL1 construct expressed under a constitutive viral CMV promoter (**b,** control), suppresses the circadian luciferase oscillation (experiment was performed 3 times). **D, E.** WT mice were kept under LD conditions and BM fluids (**D**, *n* = 11 mice per time point) or PB plasma (**E**, *n* = 9 mice per time point) were analyzed for CXCL12 content over time via Cosinor analysis (see Table 1 for circadian rhythm characteristics). **F.** BM cells of WT mice under LD conditions were harvested at ZT2 or ZT14 and chemotaxis activity towards CXCL12 was assessed, spontaneous migration served as control (*n* = 6 mice per time point). **G.** WT mice were kept under DD conditions and BM LSK cells were analyzed for CXCR4 expression via FACS analyses around the clock (*n* = 5 mice per time point). Sinusoidal function was obtained by Cosinor analysis (see Table 1 for rhythm parameters).

The interaction between the BM stroma derived chemokine CXCL12 and its receptor CXCR4 is one of the foremost retention mechanisms of immature hematopoietic cells and neutrophils [44,45]. Therefore, we sought to confirm that CXCL12 is itself subject to circadian variation and that by this mechanism circadian oscillations of hematopoietic cells are induced, as had been posited previously [19]. In silico analyses identified several putative clock gene product binding sites in the murine and human *CXCL12* promoter: eight E-boxes were identified in the murine (Figure 5A) and three E-boxes in the human promoter (Figure 5B), supporting the possibility of circadian regulation. A luciferase construct driven by the *CXCL12* promoter was tested in the murine stroma cell line NIH3T3. As a control, BMAL1 was constitutively co-expressed under a strong viral CMV promoter, in order to override cell endogenous cyclical BMAL1 expression. After synchronization of the cells with dexamethasone-supplemented medium, *CXCL12* promoter-driven luciferase expression demonstrated pronounced circadian rhythmicity, which was largely suppressed by constitutive BMAL1 overexpression (Figure 5C), indicating the circadian regulation of CXCL12 expression via cortisol and clock gene transcription factors in vitro. 

Given the possibility that organismic circadian regulation of CXCL12 expression in vivo might contribute to the cyclical changes in WBC and CFU-C, we measured the CXCL12 protein content in BM fluids and in PB plasma of WT mice around the clock. CXCL12 protein in the BM followed a significant circadian function over the course of the day (Figure 5D), along with reciprocal changes in PB CXCL12 plasma content (Figure 5E), albeit here not reaching statistical significance.

To test for potential differences in CXCL12 responsiveness as a result of circadian variation in the corresponding CXCR4 signal integration, WT mice were sacrificed at the time of peak and trough for circulating leukocytes and BM was harvested. Whole BM cells (overwhelmingly hematopoietic cells both immature and mature) recovered by flushing femurs were subjected to transwell migration assays towards a CXCL12 gradient and determination of spontaneous migration served as controls. The efficiency of CFU-C migration towards CXCL12 was the same at both time points (Figure 5F). Circadian fluctuation of progenitor and mature leukocytes was thus not dependent on a direct modulation of CXCR4 expression, nor signal integration by CXCR4, once more underscoring the notion of hematopoietic extrinsic mechanisms of blood cell oscillation. In line with this, CXCR4 surface and gene expression of immature and mature cell species in the different hematopoietic compartments was not subject to circadian regulation (exemplarily shown for CXCR4 surface expression on BM LSK cells, Figure 5G).

### 3.6. Circadian Hematopoietic Cell Oscillation is Modulated by Cortisol

**Figure 6:**
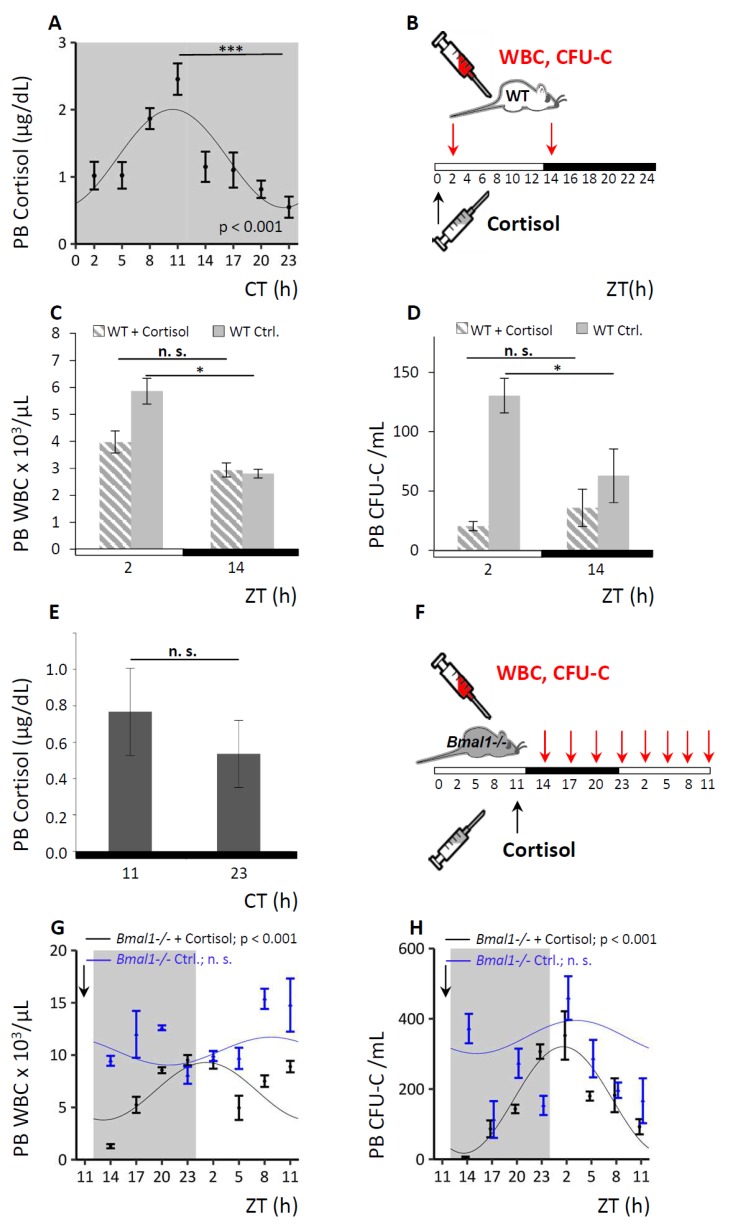
Circadian hematopoietic cell oscillation is modulated by cortisol. **A.** WT mice were held under DD conditions and endogenous PB cortisol levels were assessed over time via Cosinor analysis (*n* = 8 mice per time point). **B–D.** WT mice were analyzed under LD conditions after cortisol application. A scheme of the experimental set-up is shown in (**B**): a single dose of cortisol (in carrier fluid) or carrier fluid only (control) was injected at ZT0 (black arrow) and PB was collected at ZT2 and ZT14 (red arrows). Enumeration of PB WBC (**C**) and CFU-C counts (**D**) was performed, hatched bars indicate cortisol-treated, grey bars control treated mice (*n* = 10 mice per each group and time point). **E.** *Bmal1−/−* mice were kept under DD conditions and endogenous PB cortisol levels were assessed at ZT11 and ZT23 (*n* = 8 mice per time point). **F–H.** *Bmal1−/−* mice were analyzed under LD conditions over a period of 24 h after cortisol application. Sinusoidal function was obtained by Cosinor analysis (see Table 1 for sinusoidal rhythm parameters). A schematic experimental set-up is shown in (**F**): mice received a bolus of cortisol (in carrier fluid) or carrier fluid only (control) at ZT11 (black arrow) and PB was collected over a period of 24 h (red arrows). Enumeration of PB WBC (**G**) and CFU-C counts (**H**) was performed, black lines indicate cortisol-treated (*n* = 5–7 mice per time point), and blue lines diluent-treated *Bmal1−/−* mice (*n* = 5–15 mice per time point). Asterisks: * *p* < 0.05, *** *p* < 0.001.

The corticoliberin–corticotropin–cortisol axis is a major pathway for systemic dissemination of central circadian cues and can in fact override these when necessary [17,46]. Therefore, we hypothesized that cortisol might be involved in the transmission of circadian rhythms to the BM stroma and consequently modulate the circadian hematopoietic cell oscillation. As we were able to induce rhythmic clock gene expression via dexamethasone in NIH3T3 in vitro, the effect of changes in cortisol levels on the hematopoietic cell fluctuation in WT and *Bmal1−/−* mice was investigated in vivo. Circadian rhythmicity of endogenous cortisol levels was confirmed in the plasma of untreated WT mice, reaching peak values in the evening at CT11, i.e., three hours before trough levels of circulating CFU-C were achieved (Figure 6A). Administration of an anti-cyclical approximately physiological single dose of cortisol at the time of the natural cortisol trough at ZT0 (see Figure 6B for experimental set-up), suppressed the subsequent matutinal peak of WBC (Figure 6C) and CFU-C counts in PB (Figure 6D), affecting roughly equally all mature and immature subpopulations in WT mice (see Figures S7 and S8 for differential cell counts).

By contrast, *Bmal1−/−* mice lacked the characteristic rhythmical cortisol secretion as expected (Figure 6E). We administered a bolus injection of cortisol in *Bmal1−/−* mice at ZT11, when the peak for cortisol in WT mice would normally occur, and analyzed for PB WBC and CFU-C content around the clock (for experimental set-up see Figure 6F). Cortisol administration restored circadian rhythmicity of the WBC (Figure 6G) and CFU-C oscillation (Figure 6H) in *Bmal1−/−* mice with a significant sinusoidal curve progression after cortisol injection, unlike diluent-treated *Bmal1−/−* controls (Figure 6G,H). In the cortisol treated *Bmal1−/−* mice a distinct blood cell trough was observed three hours after the induced cortisol peak followed by a steady increase over the next 12 h, approximately resembling the circadian pattern of WT controls. The observations strongly implicate cortisol as a regulator of the circadian hematopoietic cell oscillations and further suggest that cortisol might provide cues for mature and immature cell clearance from the circulation.

## 4. Discussion

Oscillations in PB WBC of all lineages as well as immature cell counts have long been appreciated [15,16,17]. Evidence for the underlying circadian regulation was provided by the absence of rhythmical cell circulation after ablation of the central pacemaker and by clock gene mutations in mice [10,18]. However, the origin and nature of the cues conveying the circadian information from the SCN to the hematopoietic cells has been discussed controversially [43,46,47,48,49,50]. In the study presented here, we were able to confirm the circadian nature of the hematopoietic cell oscillation based on our round the clock observations in WT mice kept under stringent DD conditions along with the lack of rhythmicity found in clock gene deficient *Bmal1−/−* mice. At peak and trough a total of 1000 versus 100 CFU-C was detected in the circulation given a whole blood volume of approximately 2 mL per mouse (500 versus 50 CFU-C/mL), whereas the cumulative BM CFU-C content is approximately 800,000 cells, assuming 50,000 cells per femur, the equivalent to 1/16th of the total BM mass [51]. Therefore, although no circadian fluctuation of mature or immature cells was found in the BM compartment over the course of the day, the observed substantial changes in blood cell counts can be easily attributed to the trafficking between PB and BM, as the number of PB cells represents only a minute fraction of the total hematopoietic stem and progenitor cell (HSPC) pool. Hence, circadian cell circulations originating from redistribution between BM and PB would not noticeably alter the BM CFU-C content, due to the lack of sensitivity of available enumeration methods. Additionally, circadian hematopoietic blood cell oscillations cannot be explained by re-distribution between PB and spleen, as WBC counts in the two hematopoietic compartments oscillate in synchrony. 

In contrast to our data, remarkable diurnal changes of most hematopoietic cells in BM have been reported without equivalent changes in blood [47,48,52]. However, how such changes can be reconciled with HSPC biology is not clear, as about 50% of the total HSPC mass, suggested to disappear and re-emerge within only a few hours, can neither differentiate to a post-HSPC stage, nor leave the BM, nor die in such a short time-span. Given the overwhelmingly quiescent nature of stem cells, a replenishment of the half-empty pool within one day does not seem physiologically possible.

In our study, cell cycle analyses of mature and immature hematopoietic cells in the different hematopoietic compartments revealed no circadian variations. These observations indicate that the circadian oscillation in circulating CFU-C does not reflect larger quantitative changes in the entire immature and mature hematopoietic cell pool, instead, once more, implying a redistribution effect between PB, spleen, and other physical sites. These effects could serve a physiological purpose and represent an attempt to correct changes in the distribution of immature cells between hematopoietic compartments, for example to compensate for cell loss that occurred over the course of the day. That said, a possible contribution of very subtle cell cycle changes cannot be excluded due to insensitivity of the assay. 

By generating chimeric mice with clock gene function disrupted exclusively in the hematopoietic system, we provide first unequivocal evidence that the hematopoietic cell-autonomous clock is essentially dispensable for circadian changes in circulating blood cell numbers. Instead, hematopoietic-extrinsic cues, i.e., the BM stroma, appear to dictate the rhythmical oscillation of HSPC, as mature and immature cell concentrations in PB of mice with clock gene deficient hematopoiesis continued to oscillate in a circadian fashion in the WT environment. *Bmal1−/−* HSPC efficiently radio-protected and long-term repopulated lethally irradiated WT hosts, in which all lineages were grossly numerically normal and functionally competent. Our findings agree with and expand on the observations reported by Mendez-Ferrer et al. [19], according to which sympathetic nervous system induced rhythmical changes in the BM stroma provide the cues for the oscillations in PB cells. In contrast, using lineage-specific *Bmal1*-deletion for a subtype of monocytes, Nguyen et al. had proposed the circadian oscillations in PB to be due to the cell-autonomous clock gene expression [52]. In our study, oscillation of *Bmal1−/−* monocytes in WT hosts was quantitatively the same as that of all other mature lineages. However, the specific pattern of Ly6C-bright monocytes was not separately assessed in our experiments and functional aspects of individual mature immune cells recovered from mice reconstituted with *Bmal1−/−*BM were not specifically investigated. Mature hematopoietic cells have been shown to display circadian gene expression which drives some of their functions [21,22,53] although, as we are demonstrating, the internal clock is not responsible for the circadian oscillations in PB leukocyte counts. By contrast, HSPC themselves have no endogenous rhythm at the population level and only acquire synchronized rhythmicity during maturation [54]. 

We found the circadian oscillation of WBC and CFU-C in the circulation of humans to be inverted relative to mice, in line with observations described previously [15,16,17]. Thus, peak values were detected in the evening, onset of the resting phase, whereas trough was in the morning. Interestingly, human cells engrafted in xenotolerant NSG mice, themselves devoid of circadian rhythms, lost their circadian oscillation, confirming the role of hematopoietic-extrinsic cues for rhythmic leukocyte fluctuation in PB. In line with our data, Borkowska et al. [43] reported circadian changes in the levels of several components of the three archaic protease cascades and proposed contributory effects of the complement system to the circadian blood cell oscillation, based on absence of the rhythm in C5-deficient mice. In contrast to these observations phenotypically confirmed by us here, Zhao et al. [55] recently observed anti-cyclic fluctuations of murine and human hematopoietic cells in humanized NSG mice with amplitudes as in normal WT mice. Whether these differences can be explained by the different experimental conditions (different sources of hematopoietic cells, different light-dark cycle conditions) is not fully clear. 

The CXCL12/CXCR4 axis is one of the crucial HSPC retention mechanisms in the BM and has been implicated as a hematopoietic extrinsic signal controlling circadian HSPC redistribution [44,45]. Pronounced diurnal variations of CXCL12 protein levels in the BM stroma as well as leukocyte CXCR4 expression have been reported [56,57,58]. In vitro data presented herein provide evidence for a circadian regulation of *CXCL12* via cortisol and clock gene transcription factors. In vivo analyses confirmed CXCL12 protein in the BM in agreement with the work by Mendez-Ferrer et al. [19] and revealed an inverse oscillation of CXCL12 plasma protein levels, albeit, due to the relatively flat amplitude of PB plasma levels, here not reaching statistical significance.

A potential role of cortisol in circadian cell oscillations has been considered [46,58]. In agreement with its function as a conveyor of central circadian rhythms to peripheral tissues [59], we provide evidence for regulation of clock gene expression via glucocorticoids in vitro and suggest that plasma cortisol levels and blood cell fluctuation might be directly functionally related in vivo. In mice, endogenous vespertine cortisol peak preceded the PB cell count nadir by about three hours. The application of a single cortisol dose, inverted to the physiological plasma peak, suppressed the matutinal leukocyte acrophase in WT mice, whereas physiological cortisol administration in *Bmal1-/-* mice restored rhythmic HSPC oscillation. These observations suggest that cortisol represents a signal for mature and immature cell clearance from the circulation. Thus, peak cell numbers are not a consequence of increased release from the BM but rather the trough is achieved due to improved clearance from the circulation in response to rising cortisol levels. This hypothesis is supported by earlier work on lymphocytes, where adrenalectomy in WT mice ablated rhythmic lymphocyte circulation, whereas cortisol bolus injections induced lymphocyte clearance from PB [17]. Cortisol-mediated lymphocyte homing to BM has been suggested more recently [56,60,61] and the reported circadian changes in blood lymphocyte counts are in agreement with ours. 

As in *Bmal1−/−* mice not only the central but also the peripheral clock is absent, the induction of rhythm via cortisol administration might appear to be in contrast to the proposed mechanism of (stromal) *Cxcl12* as a direct BMAL1 target. However, published work demonstrates that in clock-gene-deficient organs of animals with an intact master SCN, superordinate circadian oscillators are able to rescue or compensate for genetic defects affecting phase and period of peripheral clocks [2,4]. In addition, glucocorticoids have been shown to directly induce clock gene expression by resetting the phase of *Per1* [1,59]. Application of cortisol in *Bmal1−/−* mice could therefore restore "half of the rhythm" via direct regulation of PER1 expression in peripheral tissues. The "rhythm" is then maintained until the lack of counter regulation by its missing antagonist BMAL1 becomes apparent and the oscillation abates.

## 5. Conclusions

In summary, we provide first unequivocal evidence ruling out a cell-autonomous role for clock genes in the circadian oscillation of hematopoietic cells and further demonstrate that the marked changes in the peripheral hematopoietic organs, blood, and spleen are not accompanied by similar activity in BM. Cell cycle analyses in hematopoietic cells point towards redistribution of HSPC from the BM, as opposed to cell cycle-dependent qualitative or quantitative changes, to be responsible for peripheral cell count oscillations. Rhythmicity of CXCL12 expression was confirmed with cortisol involved in its regulation in vitro as well as mediating the circadian HSPC release from the BM in vivo.

## Figures and Tables

**Table 1:** Circadian rhythm parameters.

Figure	Parameter	MESOR	Amplitude	Acrophase (hrs)	Significance (Global *p*, FDR corrected)
1A	PB WBC × 10^3^/µL	6.3	3.3	−1.2	<0.001
1C	PB CFU-C/mL	154	69	−0.9	<0.001
1D	PB WBC × 10^3^/µL	7.4	4.2	−1.4	<0.001
1F	PB CFU-C/mL	253	237	−1.4	<0.001
1J	WBC × 10^6^/femur	15.7	0.9	−2.4	n. s.
2A	PB WBC × 10^3^/µL	10.6	1.4	−2.4	n. s.
2C	PB CFU-C × 10^2^/mL	3.4	0.48	−0.54	n. s.
2G	WBC × 10^6^/femur	20	0.78	−0.62	n. s.
2H	LSK × 10^4^/femur	7.8	0.85	−0.68	n. s.
4B	WT BM/WT: PB WBC × 10^3^/µL	10	5.2	−1.5	<0.001
	*Bmal1−/−* BM/WT: PB × 10^3^/µL	11	4.3	−1.0	<0.001
4D	WT BM/WT: PB CFU-C/mL	193	75	−1.5	<0.01
4D	*Bmal1−/−* BM/WT: PB CFU-C/mL	196	78	−1.3	<0.05
5D	BM CXCL12 (ng/mL)	44	7.8	−1.3	<0.05
5E	PB CXCL12 (ng/mL)	0.92	0.09	−4.4	n. s.
5G	BM LSK cells, MFI of CXCR4	830	74	−2.4	n. s.
6A	PB Cortisol (µg/dL)	1.3	0.73	−2.7	<0.001
6G	*Bmal1−/−* + Cortisol: PB WBC × 10^3^/µL	6.5	2.8	−0.30	<0.001
	*Bmal1−/−* Ctrl.: PB WBC × 10^3^/µL	10.4	1.3	−2.3	n. s.
6H	*Bmal1−/−* + Cortisol: PB CFU-C/mL	169	152	−0.41	<0.001
	*Bmal1−/−* Ctrl.: PB CFU-C/mL	348	47	−0.82	n. s.
S1	B cells × 10^3^/µL	4.0	2.8	−1.6	<0.001
	Granulocytes × 10^3^/µL	0.80	0.45	−0.56	<0.01
	Monocytes × 10^3^/µL	0.45	0.22	−1.5	<0.001
	T cells × 10^3^/µL	3.0	1.4	−1.4	<0.001

Sinusoidal function was analyzed by the Cosinor method. MESOR indicates average value during 24-h period; amplitude, difference between estimate maximum/minimum and MESOR; acrophase, time of day of the maximum; significance, *p-*value of the zero amplitude test, FDR corrected.

## Supplementary Materials

The following are available online at https://www.mdpi.com/2073-4409/8/9/1033/s1, Figures S1-S8: Supplements.
