# Supplementary material for: Hematopoietic-Extrinsic Cues Dictate Circadian Redistribution of Mature and Immature Hematopoietic Cells in Blood and Spleen
Source: Cells. 2019 Sep 5;8(9):1033. doi: 10.3390/cells8091033 (PMC6769956; doi:10.3390/cells8091033)
Supplement: Supplementary file 1 [file cells-08-01033-s001.pdf]

Supplements

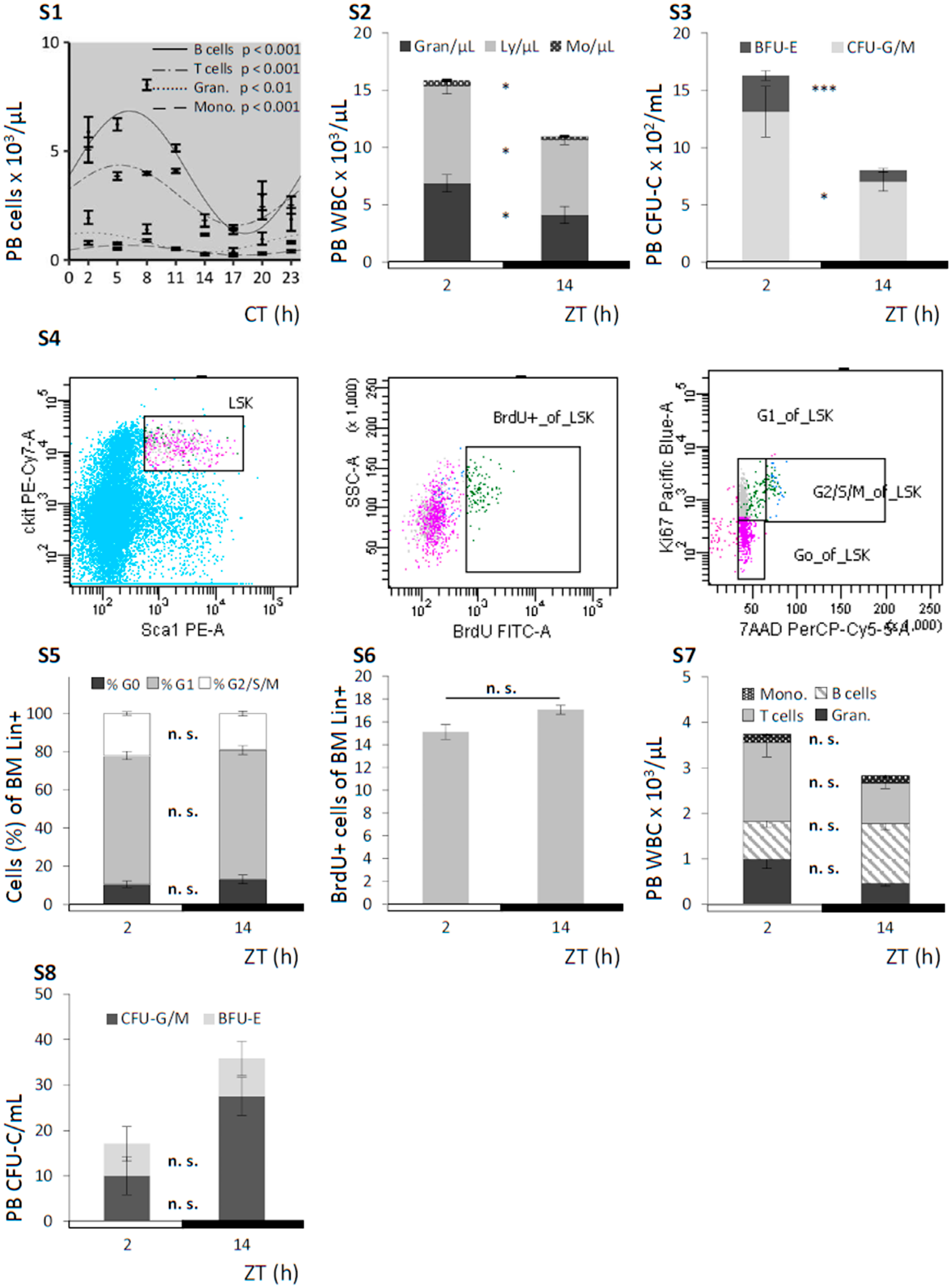

S1. Hematopoietic cell concentrations in PB. Cosinor analysis was performed for leukocyte subpopulations of WT mice under DD conditions, for circadian rhythm characteristics see Table 1 (n = 8 – 16 mice per time point).

S2, S3. Hematopoietic cell oscillation under AMD3100. WT mice received AMD3100 ZT1 and ZT13 mature (**S2**) and immature (**S3**) leukocyte subpopulations in PB were assessed at ZT2 and ZT14 (n = 9 mice per time point). Asterisks: * $P < 0.05$, ** $P < 0.01$, *** $P < 0.001$.

S4-S6. Cell cycle activity of BM cells. S4 displays the gating strategy for LSK cell cycle analyses via anti-BrdU staining after short-term BrdU-pulsing or via intracellular Ki67/7-AAD staining. Cell cycle activity of BM Lin+ cells was assessed by Ki67/7-AAD co-staining (**S5**, G0 phase depicted in dark grey, G1 phase in light grey and G2/S/M phase in white) or BrdU incorporation (**S6**) at ZT2 and ZT14 after 3-hour BrdU pulsing (n = 6 mice per time point).

S7, S8. Hematopoietic cell oscillation is modulated by cortisol. WT mice under LD conditions received a single dose of cortisol at ZT0 and PB was collected at ZT2 and ZT14. Enumeration of PB WBC (**S7**) and CFU-C (**S8**) subpopulations was performed (n = 10 mice per time point).
